# Comparative analysis of lower limb biomechanics during unilateral drop jump landings on even and medially inclined surfaces

**DOI:** 10.1371/journal.pone.0322562

**Published:** 2025-05-02

**Authors:** Ahmed Dami, Eléna Payen, Pier-Luc Isabelle, Nader Farahpour, Gabriel Moisan

**Affiliations:** 1 Groupe de Recherche sur les Affections Neuromusculosquelettiques (GRAN), Université du Québec à Trois-Rivières, Trois-Rivières, Québec, Canada; 2 Department of Anatomy, Université du Québec à Trois-Rivières, Trois-Rivières, Québec, Canada; 3 Department of Human Kinetics, Université du Québec à Trois-Rivières, Trois-Rivières, Québec, Canada; 4 Department of Sport Biomechanics, Faculty of Sport Sciences, Bu-Ali Sina University, Hamedan, Iran; University of Tehran, IRAN, ISLAMIC REPUBLIC OF

## Abstract

**Background/purpose:**

Lower limbs biomechanics during unilateral jump landing, a common sports maneuver, is widely studied in research. Most studies used an even surface which may not be ecologically valid in sports contexts. There is a need to explore the lower limbs biomechanics during landing on other, more challenging, surfaces. The purpose of this study was to investigate the lower limb kinematic and kinetic differences during unilateral drop jump landing from a 30 cm platform on even (DROP) and medially inclined (WEDGE) surfaces.

**Methods:**

Fifteen healthy participants were recruited to take part in this laboratory-based cross-sectional study. The experimental protocol involved comparing hip, knee, ankle and midfoot angles, moments, and power between DROP and WEDGE.

**Results:**

Main kinematic findings were that during WEDGE, midfoot inversion angles were smaller, and ankle eversion and plantarflexion, knee abduction and internal rotation and hip abduction angles were greater compared to DROP. Main kinetic results were that during WEDGE, midfoot inversion and adduction moments, ankle inversion moments, knee adduction moments, hip adduction and internal rotation moments, midfoot and ankle power were greater and ankle plantarflexion and adduction moments and knee internal rotation moments were smaller compared to DROP.

**Conclusion:**

These adaptations highlight the intricate interaction between surface inclination and joint movements. This study’s results not only contribute valuable insights into the biomechanics of landing on inclined surfaces but also lays the foundation for future research that can refine injury prevention strategies, optimize training protocols, and enhance the overall performance and safety of athletes across various sports.

## Introduction

Landing from a jump is common in daily activities, but its significance is particularly pronounced within the context of sports maneuvers. Effective execution of jump landings is crucial to be successful and performant across various sports, particularly those involving jumps, such as basketball, volleyball, and ski jumping. The forces encountered during jump landing can be substantial, reaching up to 10 times an individual’s body weight per second [1]. The association between jump landing tasks demanding rapid force dampening and increased risk of non-contact lower limb injuries in sports has been well established [2,3].

The interaction between the human neuromusculoskeletal system and external conditions (e.g., the landing surface), plays a pivotal role in landing performance outcomes, encompassing key parameters such as lower extremity kinematics, kinetics, and electromyographic responses [4–8]. A prominent focus within landing studies is the unilateral drop jump, a widely investigated landing task that is prevalent in many sports. Athletes routinely execute isolated landings on a single leg without subsequent motion. Furthermore, these unilateral drop jump landings constitute integral components of training and exercise regimens, enhancing their significance in research investigations aimed at uncovering injury risk factors, performance metrics, and the ability to seamlessly reintegrate into sporting engagements [5–7,9,10].

Unilateral drop jump landings are also commonly investigated tasks to identify biomechanical deficits associated with musculoskeletal disorders, such as chronic ankle instability [5–7] and anterior cruciate ligament injuries [9,10]. Furthermore, they serve as a valuable platform for evaluating the potential of biomechanical enhancements through the utilization of external aids, such as foot orthoses [8,11], surgical interventions [10], or neuromuscular training [12]. To dampen the impact during unilateral drop jump landing, individuals use a combination of preprogrammed central control (feedforward mechanism) and reflex mechanisms based on the task requirements (feedback mechanism). Before impact, individuals preactivate their hip, thigh and shank muscles to stiffen the lower limb prior to ground contact [4,5]. Shortly after the impact, large ground reaction forces as high as 2.5 times body weight, must be dampened through coordinated lower limbs movements [4,13]. To mitigate the impact, the ankle joint undergoes dorsiflexion and eversion, the knee joint flexes, abducts and internally rotates, and the hip joint adducts and internally rotates [4,5,7]. These lower limb movements significantly contribute to ankle plantarflexion and knee internal extension moments [4,6]. Lower limbs’ joints flexion and rotation reduce the axial stiffness of the entire body, forming a highly effective mechanism to limit ground reaction forces and accelerations during impacts [14]. The foot medial longitudinal arch also contributes to attenuating impacts by lowering and accumulating kinetic energy during landing [15].

Most previous studies exploring lower limb biomechanics during unilateral jump landing used an even surface which may not accurately reflect ecological conditions encountered in sports and training scenarios. Exploring lower-limb biomechanics during more challenging jump landing tasks is essential to improve clinicians’ and researchers’ understanding of the lower limb biomechanics. One of these tasks are unilateral drop jump landing on inclined surfaces [4,6,8,11,16–19]. Moisan et al. [4] reported that landing on a 25 degree laterally inclined surface decreased ankle dorsiflexion and internal rotation and increased ankle inversion compared to landing on a level surface in individuals with chronic ankle instability. They also reported greater ankle dorsiflexion and smaller ankle inversion and internal rotation moments, smaller knee adduction moments and internal rotation moments during unilateral drop jump landing on an inclined surface when compared to a level surface. However, even though studying landing on a laterally inclined surface is relevant to individuals at risk of sustaining lateral ankle injuries (e.g., chronic ankle instability), it is less relevant for those with foot and medial ankle musculoskeletal disorders (e.g., plantar fasciopathy, posterior tibialis tendon dysfunction). Thus, it is crucial to study how one adapts to unilaterally landing on a medially inclined surface, which may place substantially more stress on the foot and medial ankle structures and proximal joints [8,18,19]. Also, further explorations are needed to better understand the foot biomechanics when landing on an inclined surface, which is a major gap in the knowledge base.

This study aimed to fill this gap by investigating lower limb kinematic and kinetic differences during unilateral drop jump landing on both even and medially inclined surfaces in healthy individuals. Our hypotheses were that landing on a medially inclined surface would induce greater foot and ankle pronation, midfoot dorsiflexion and abduction, knee and hip internal rotation and ankle inversion moments as well as greater midfoot and ankle power absorption. By comprehensively assessing how landing on distinct surfaces modulates lower limb biomechanics, this study not only will enhance our understanding of the biomechanical implications during training and sports but also contributes to a more comprehensive evaluation of injury risks in athletes. These insights are invaluable for designing targeted, effective, and safer training programs that align with the diverse demands of athletic performance.

## Materials and methods

### Participants

Fifteen participants were recruited between November 3rd 2022 and August 14th 2023 to take part in this cross-sectional descriptive, laboratory-based study. As no previous study compared the lower limb biomechanics during unilateral drop landing on a 10 degree medially inclined and even surfaces, we were unable to calculate the necessary sample size due to the unavailability of required inputs for the sample size calculation formula. We chose a convenience sample of 15 participants based on sample sizes of previously published studies on landing biomechanics [4,8,20]. However, we analyzed the data for the variables of primary interest of all participants of the convenience sample. Given that the statistical power was >80% for most of these variables, we considered our sample size sufficient to address our study objectives.

Participants were recruited among the staff and students of Université du Québec à Trois-Rivières (UQTR), Canada, by means of referral from the outpatient podiatry clinic, via social media advertisements or database of our research group. To be included in this study, potential pain-free participants needed to be aged between 18 and 45 years. The exclusion criteria were to have any neuromusculoskeletal disorders, structural abnormalities in the lower limbs, history of musculoskeletal surgery in the lower limbs, current injuries that could influence foot function, pregnancy, or a history of lower limb injuries within the past three months prior to data collection. All participants provided their informed written consent to a study protocol approved by the UQTR Ethics Committee (CER-21-283-07.01).

### Instrumentation

Biomechanical analyses involved measuring the kinematics of the dominant lower limb using a three-dimensional passive motion analysis system (OptiTrack; Natural Point, Corvallis, OR, USA) at a sampling rate of 200 Hz. The modified Oxford foot model was employed for this purpose [21]. According to this marker set, anatomical markers were positioned on the bilateral anterior superior iliac spines, bilateral posterior superior iliac spines, bilateral greater trochanter, medial and lateral femoral epicondyles, medial malleolus and posterior inferior of the calcaneus during a calibration trial and then removed during dynamic trials. Participants performed the experimental protocol wearing standardized sports shoes of their foot size (Athletic Works, Model: Rupert, Bentonville, AR, USA). To attach the markers on the foot, a hole was created for each marker in the shoe. To track the pelvis, thigh and shank segments, 4-marker clusters were affixed to the sacrum and the mid anterolateral region of the thigh and leg, respectively. For the hindfoot, a 3-marker cluster composed of a triad plastic wand affixed on a small, curved plastic base (20×25 mm) was firmly attached to the posterior superior aspect of the calcaneal tuberosity through a hole in the shoe’s heel with athletic tape [4,22,23]. Ground reaction forces were recorded at a sampling rate of 1000 Hz with an in-ground force plate (AMTI, Watertown, MA, USA) embedded in the middle of the calibrated space. Force plate and video-camera systems were synchronized using Motive software.

### Protocol

**Fig 1:**
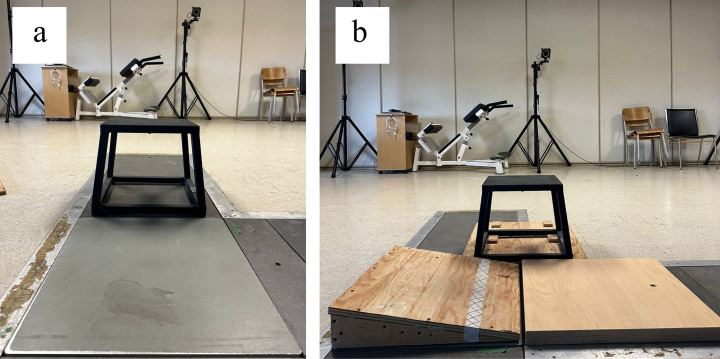
A) DROP and B) WEDGE surface.

Before undertaking the biomechanical analysis, participants’ characteristics including age, sex, mass, height, Foot Posture Index (to quantify foot morphology) [24] and supination resistance (to quantify the force required to supinate the foot) [25] scores were registered (Table 1). Then, a calibration trial was recorded for all participants following which, five trials of two tasks were completed in a random order: unilateral drop landing on a stable level surface (DROP) and unilateral drop landing on a 10 degree medially inclined surface (WEDGE). During DROP and WEDGE conditions, participants positioned themselves on a 30 cm high platform (Fig 1). Then, they propelled themselves forward with the non-dominant limb and landed on the surface with the dominant limb while keeping the other limb raised off the ground. During DROP, participants landed on the center of the force plate whereas they landed on the center of a 10 degree medially inclined wood surface placed over the force plate during WEDGE (Fig 1). Participants were asked to face forward, place their hands on their waist and stay in balance for at least two seconds after landing on the surface. Trials were rejected and retaken when participants lost balance, used their hands to restore balance and when their foot moved on the surface after landing. Prior to registering data, participants were given 5–10 minutes of familiarization to get acclimatized with the experimental protocol.

**Table 1:** Demographic data.

Variable	Value*
Sex (No. of males/females)	4/11
Age (y)	25.2 (5.5)
Mass (kg)	71.2 (19.3)
Height (m)	1.65 (0.10)
Foot Posture Index	7.9 (2.3)
Supination resistance (%BW)	12.5 (3.0)

Values indicate means (SD) unless otherwise stated. The Foot Posture Index and Supination resistance scores refer to the dominant limb.

#### Data processing.

Kinematic and kinetic data were imported and processed using Visual 3D software (version 6.01.36; C-Motion, Inc). Kinematic markers trajectories were filtered at 6 Hz and force plate data at 20 Hz with a zero-lag, fourth-order, low-pass Butterworth filter [8]. Local coordinate systems of the forefoot, hindfoot, ankle, knee and hip were defined. Midfoot, ankle, knee and hip angles were computed with an X-Y-Z order of rotations [8,26]. Midfoot angles were computed as the angle between the forefoot and the hindfoot segments [27–29]. Midfoot, ankle, knee and hip internal moments were calculated using inverse dynamics (synchronized joint kinematics/ground reaction forces and anthropometric data), normalized to body mass, and resolved in the proximal segment coordinate system. Joint power was calculated as the product of the net internal joint moment and joint angular velocity. Midfoot moments and power were computed for the forefoot resolved in the hindfoot segment coordinate system during the epoch for which only the forefoot was in contact with the surface, and not the hindfoot [27–30]. Kinematic and kinetic data were resampled to 100% of the landing phase with 0% representing the initial contact with the surface and 100% corresponding to maximal knee extension following landing except for the midfoot kinetic data which were normalized from initial contact (0%) to heel contact (100%) [8].

### Analysis

Kinematic and kinetic data were compared across conditions for each percentage of the landing phase, including the mean of all trials, using statistical parametric mapping method [31]. First, the distribution of the data was evaluated using spm1d.stats.normality.ttest_paired function. When data was normally distributed, spm1d.stats.ttest_paired function was used to compare the dependent variables across conditions. When data was not normally distributed, spm1d.stats.nonparam.ttest_paired was used. When the SPM(t) and SnPM(t) curves crossed the threshold of α = 5% for the biomechanical outcomes, suprathreshold clusters were created, indicating significant differences between DROP and WEDGE in a specific location of the landing phase. All analyses were conducted using the open-access SPM1D code (www.spm1d.org) with Matlab R2023a (The Mathworks Inc., Boston, MA, USA).

## Results

**Fig 2:**
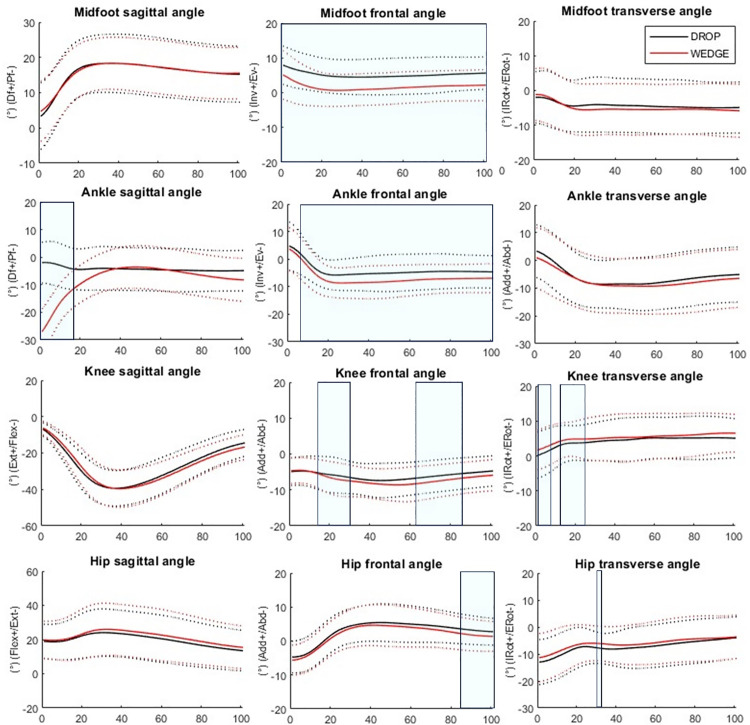
Joint angles differences between DROP (black) and WEDGE (red). Df = dorsiflexion, Pf = plantarflexion, Inv = inversion, Ev = eversion, IRot = internal rotation, ERot = external rotation, Abd = abduction, Add = adduction, Flex = flexion, Ext = extension. Solid lines = mean trajectories, Dotted lines = standard deviation trajectories. Significant differences between conditions are indicated in the shadowed regions.

During WEDGE, midfoot inversion was smaller from 0 to 100% of the landing phase (%LP) (*p* = 0.010), ankle eversion was greater from 7 to 100%LP (*p* = 0.010), ankle plantarflexion was greater from 0 to 18%LP (*p* = 0.010), knee abduction angle was greater from 18 to 30%LP (*p* = 0.018) and from 64 to 85%LP (*p* = 0.002), knee internal rotation was greater from 1 to 8%LP (*p* = 0.027) and from 14 to 25%LP (*p* = 0.011), hip abduction was greater from 85 to 100%LP (*p* = 0.027) and hip external rotation was smaller from 34 to 36%LP (*p* = 0.048) compared to DROP. No other significant differences were observed (Fig 2). All standard deviations can be observed on Fig 2.

**Fig 3:**
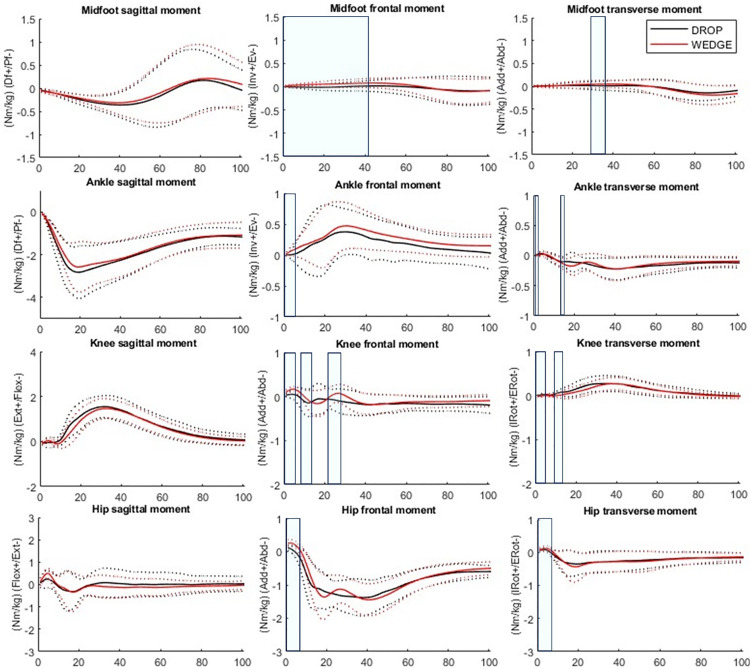
Joint moments differences between DROP (black) and WEDGE (red). Df = dorsiflexion, Pf = plantarflexion, Inv = inversion, Ev = eversion, IRot = internal rotation, ERot = external rotation, Abd = abduction, Add = adduction, Flex = flexion, Ext = extension. Solid lines = mean trajectories, Dotted lines = standard deviation trajectories. Significant differences between conditions are indicated in the shadowed regions.

**Fig 4:**
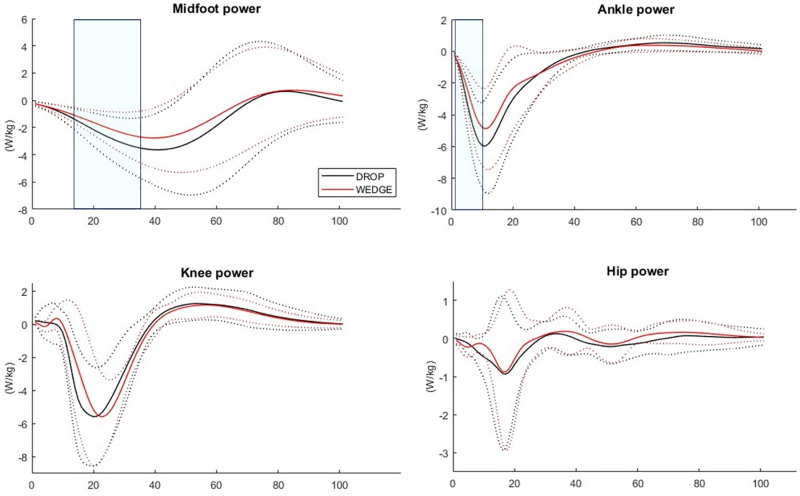
Joint power differences between DROP (black) and WEDGE (red). Df = dorsiflexion, Pf = plantarflexion, Inv = inversion, Ev = eversion, IRot = internal rotation, ERot = external rotation, Abd = abduction, Add = adduction, Flex = flexion, Ext = extension. Solid lines = mean trajectories, Dotted lines = standard deviation trajectories. Significant differences between conditions are indicated in the shadowed regions.

During WEDGE, midfoot inversion and adduction moments were greater from 0 to 41% (*p* < 0.001) and from 28 to 36% of the initial contact phase (*p* = 0.030), respectively, compared to DROP. Ankle inversion moments were greater from 0 to 5%LP (*p* = 0.028) and ankle adduction moments were smaller from 1 to 2%LP (*p* = 0.020) and from 14 to 16%LP (*p* = 0.020) compared to DROP. Knee adduction moments were greater from 0 to 4%LP (*p* = 0.032) and from 25 to 29%LP (*p* = 0.038), knee internal rotation moments were smaller from 0 to 3%LP (*p* = 0.038) from 9 to 12%LP (*p* = 0.038) and from 22 to 26%LP (*p* = 0.030), hip adduction moments were greater from 0 to 6%LP (*p* = 0.013) and hip internal rotation moment were greater from 0 to 6%LP (*p* = 0.010) during WEDGE compared to DROP. Midfoot power was greater from 15 to 35%LP (*p* = 0.004) and ankle power was greater from 2 to 9%LP (*p* = 0.010) during WEDGE. No other significant differences were observed (Figs 3 and 4). All standard deviations can be observed on Figs 3 and 4.

## Discussion

Most previous studies investigated the lower limbs biomechanics during landing on a flat surface or a laterally inclined surface. However, it is crucial to study how one adapts to unilaterally landing on a medially inclined surface, which may place substantially more stress on the foot and medial ankle structures and proximal joints [8,18,19]. Thus, this study aimed to investigate lower limb kinematic and kinetic differences during unilateral drop jump landing on both even and medially inclined surfaces. Our hypotheses were that medially inclined surface landings would induce greater foot and ankle pronation, midfoot dorsiflexion and abduction, knee and hip internal rotation and ankle inversion moments as well as greater midfoot and ankle power absorption.

First, the biomechanical patterns observed in the lower limbs during DROP closely resembled those documented in previous studies. Specifically, ankle eversion was noted to trigger a sequence of events involving knee flexion, abduction, and internal rotation, subsequently followed by hip adduction and internal rotation, as reported in previous research [4,5,7]. These sequential movements were found to result in significant ankle plantarflexion and knee internal extension moments [4,6], with the additional observation that the midfoot segment played a role in mitigating impacts through dorsiflexion [15].

During WEDGE, as anticipated, participants exhibited greater ankle eversion during landing. Interestingly, despite the surface medial inclination of 10^o^, the maximal difference in ankle eversion angles between DROP and WEDGE was merely 3.2^o^. This observation suggests that proximal and distal joints assisted the ankle joint to obtain the requisite position for the foot to be plantigrade with the surface. This result is similar to those of Moisan et al. [4] who reported a 6.3^o^ difference in ankle inversion during landing on a 25 degree laterally inclined surface compared to an even surface. In our study, a portion of the distal compensations came from the midfoot as highlighted by the increased midfoot inversion. The combination of greater ankle eversion and greater midfoot inversion suggest flatter feet during WEDGE [8]. These adaptive changes in kinematics, particularly during the initial contact phase, seem to exert greater stress upon the ankle and midfoot segments, as indicated by the greater ankle inversion and midfoot inversion and adduction moments during WEDGE. However, as the ankle joint is plantarflexed when landing on the inclined surface, less ankle and midfoot sagittal power needs to be absorbed by these joints. Overall, greater ankle eversion angles and inversion moments increase the physiological demand on the medial and plantar structures such as the ankle deltoid ligaments, the posterior tibial muscle and the plantar fascia. These insights hold potential significance in the realm of musculoskeletal disorders, such as posterior tibialis tendon dysfunction and plantar fasciopathy.

Furthermore, the greater ankle and midfoot frontal movements were found to trigger compensatory adjustments in the knee and hip. Foot and ankle pronation is associated with knee internal rotation and abduction and hip internal rotation. As anticipated, we found greater hip and knee internal rotation and knee abduction, suggesting a pronated pattern of the kinetic chain. Although we did not observe differences in knee and hip power, alterations in knee and hip kinematics resulted in corresponding changes in joint strains, as indicated by alterations in moments. Interestingly, most biomechanical differences occurred at or shortly after the impact (Figs 2 and 3), which could have significant implications in sports as many musculoskeletal injuries occur during this period [2,3].

The findings of this study have significant clinical implications as athletes must often land and stabilize on challenging surfaces in sport-specific contexts. Understanding how landing on different surfaces influences lower limb biomechanics during unilateral drop jump landings provides valuable insights for injury prevention and rehabilitation and training strategies. By examining the responses of the lower limb to medially inclined surfaces, clinicians can tailor interventions to better address the specific demands athletes face in various sports scenarios. Individuals with foot and medial ankle musculoskeletal disorders, such as plantar fasciopathy or posterior tibialis tendon dysfunction, may benefit from interventions that account for the increased stress associated with landing on medially inclined surfaces. Thus, this study aids in the development of more effective injury management strategies that align with athletes’ unique anatomical and biomechanical considerations.

The first limitation of our study is that data collection sessions took place in a highly controlled environment which may not entirely mirror the ecological contexts in which athletes routinely execute unilateral jump landings. This controlled setting, while beneficial for isolating dependent variables, may not fully encapsulate the multifaceted conditions athletes encounter during their athletic tasks. Simpson et al. [32] highlighted the presence of anticipatory motor control strategies when participants knew they will land on an inclined surface. This underscores the potential influence of cognitive factors on landing strategies, particularly when confronted with atypical terrains or landing scenarios. Also, in sports activities, jumping height can be higher than 30 cm. Thus, if landing from higher than 30 cm, athletes may present additional biomechanical changes. The second limitation is the sex distribution of participants, including more females than males (11 females, 4 males). Our findings may therefore be more generalizable to females. A more balanced sex distribution could enhance the broader applicability of our results. The third limitation is that only young asymptomatic participants were included. While our mean age of 25.2 years aligns with university-level athletes, the findings of our study may not be directly transferable to older athletes or those with musculoskeletal injuries. Extending our investigations to encompass a broader age spectrum and individuals with varying injury profiles could offer a more comprehensive understanding of the implications across different athlete cohorts. The fourth limitation is the lack of control over the foot/shank muscle strength. It is not clear how muscle strength may influence the foot kinematics and kinetics when landing on a medially inclined surface. However, the utilization of the same group of participants for landings on two distinct surfaces enables us to infer that the observed variations can be attributed to the divergence in surface inclinations. The fifth limitation is that the Oxford foot model, used to assess foot kinematics, is sensitive to soft tissue artifacts [33]. Additionally, this three-segment model simplifies midfoot angles, reducing accuracy in evaluating complex foot kinematics compared to a four-segment model like the Rizzoli foot model [34]. However, due to potential marker tracking loss inside the shoes and the risk of compromising their structural integrity by adding more holes to accommodate the Rizzoli foot model, the Oxford foot model was chosen despite its limitations, given its excellent within-session reliability [35]. Considering the large standard deviations for some midfoot variables, readers should interpret these results with caution.

## Conclusions

Medially inclined surfaces induced significant kinematic and kinetics changes. This was evidenced by several key biomechanical indicators such as increased midfoot inversion, ankle eversion, knee abduction and internal rotation and hip internal rotation angles, ankle inversion and knee adduction moments and decreased midfoot and ankle power absorption. These adaptations, especially during the initial impact, highlight the intricate interplay between surface inclination and joint movements. This study not only contributes valuable insights into the biomechanics of landing on inclined surfaces but also lays the foundation for future research that can refine injury prevention strategies, optimize training protocols, and enhance the overall performance and safety of athletes across various sports disciplines.
